# Low Behavioral Intention to Use Any Type of HIV Testing and HIV Self-Testing among Migrant Male Factory Workers Who Are at High Risk of HIV Infection in China: A Secondary Data Analysis

**DOI:** 10.3390/ijerph20065029

**Published:** 2023-03-13

**Authors:** Kechun Zhang, Paul Shing-fong Chan, Xinyue Li, Yuan Fang, Yong Cai, Huachun Zou, Bolin Cao, He Cao, Tian Hu, Yaqi Chen, Zixin Wang

**Affiliations:** 1 Longhua District Center for Disease Control and Prevention, Shenzhen 518110, China; 2 Jockey Club School of Public Health and Primary Care, The Chinese University of Hong Kong, Hong Kong, China; 3 Department of Health and Physical Education, The Education University of Hong Kong, Hong Kong, China; 4 School of Public Health, Shanghai Jiaotong University School of Medicine, Shanghai 200025, China; 5 School of Public Health (Shenzhen), Sun Yat-sen University, Shenzhen 518107, China; 6 Kirby Institute, University of New South Wales, Sydney, NSW 2052, Australia; 7 School of Media and Communication, Shenzhen University, Shenzhen 518060, China

**Keywords:** HIV testing, HIV self-testing, migrant workers, rural-to-urban migrants, high-risk sexual behavior, behavioral intention, Health Belief Model

## Abstract

This study investigated the prevalence of and factors associated with behavioral intention to take up any type of HIV testing and HIV self-testing (HIVST) in the next six months among male migrant workers, who were at high risk of HIV infection, in Shenzhen, China. This was a secondary data analysis. A total of 363 subjects who had sexual intercourse with non-regular female sex partners and/or female sex workers in the past six months were selected. Logistic regression models were fitted for data analysis. About 16.5% of participants reported having used HIV testing in their lifetime and 12.7% for HIVST. Among the participants, 25.6% and 23.7% intended to take up any type of HIV testing and HIVST in the next six months, respectively. Significant factors associated with the behavioral intention to take up HIV testing and HIVST included individual-level factors based of the Health Belief Model (e.g., perceived benefit, perceived cue to action, perceived self-efficacy) and interpersonal-level factors (e.g., frequency of exposure to health-related content or HIV and STI-related content on short video apps). This study provided practical implications for designing interventions to increase the uptake of HIV testing and HIVST among migrant workers.

## 1. Introduction

Human immunodeficiency virus (HIV) infection is a public health issue in China, with 1.1 million people living with HIV (PLWH) and 351,000 associated deaths reported by the end of 2020 [1]. The predominant transmission route of HIV infection in China has changed from injection drug use and/or contaminated plasma to sexual transmission in the last decade [2]. In 2020, heterosexual transmissions accounted for 74.2% of all cases, an increase from 48.3% in 2009 [3]. Studies showed that about three-quarters of HIV infections did not involve sexual relations with spouses. Having sex with non-regular sex partners (NRPs) contributed to heterosexual transmission including commercial heterosexual and non-marital non-commercial sexual contact [4,5,6,7,8,9]. According to the China Center for Disease Control and Prevention (CDC), people engaging in commercial or non-marital and non-commercial heterosexual contact are at high risk of HIV infection [10]. These groups are recommended to receive regular HIV testing [10].

HIV prevalence in migrants is reported to be higher than that in the general male population both internationally [11] and locally [12]. Migration is considered a significant contributor to the spread of HIV [6,13,14]. A meta-analysis showed that 53.4% of the HIV-infected individuals in urban areas were migrant workers [15]. Moreover, the prevalence of HIV infections among migrants was 1.8 times higher than that of rural residents who did not migrate to cities and was associated with high-risk sexual behavior [8,14,16]. There were 376 million migrants in 2020 in China [17]. It is likely that the migrant population spreads the HIV epidemic from one region of the country to another due to its large size [6]. From 2016 to 2018, among the 2925 newly reported HIV/AIDS cases in Shenzhen, the largest migrant city in Guangdong, 79.5% were males and 83.7% were migrants [18]. Over 80% of the migrants in Shenzhen work in factories and 40% have two or more sexual partners [13,19]. Being separated from their spouses or family members, social constraints may be less important to migrants. This also creates anonymity, which makes them have a more open attitude towards sexual behaviors and thus more likely to engage in high-risk sexual behaviors [20,21]. Male factory workers engaging in sexual contact with non-regular female partners (NRPs) and/or female sex workers (FSWs) are more at risk of sexually transmitted infections than those with regular partners (RPs) [22]. They are mainly clients of FSWs with high HIV prevalence and low HIV testing uptake [23]. The high HIV prevalence among migrant workers and their seasonal return to villages during the Chinese Spring Festival raise significant concerns for HIV transmission.

Ensuring high coverage of HIV testing (i.e., >90%) among the at-risk population is the first and most crucial step to achieve the 90–90–90 targets established by the Joint United Nations Programme on HIV/AIDS (UNAIDS) [24]. However, there are no official data on the uptake of HIV testing among the high-risk migrant population. The UNAIDS has declared the migrant population as one of the key populations, and millions are unaware of their HIV sero-status [25]. Since 2003, China has made a lot of efforts in response to the HIV epidemic. This includes free access to HIV testing and counseling, free medication for people with AIDS, and other services [26,27]. Progress on facility-based HIV testing can be seen in the increasing number of sites for voluntary counseling and testing (VCT) from 6077 in 2008 to 11,319 in 2020 [3]. In addition, medical institutions have promoted opt-out testing [3]. The feasibility of HIV self-testing (HIVST) has been reviewed in high-risk populations, including men who have sex with men (MSM) in China [28]. HIVST kits may be obtained free of charge from local communities and for less than 20–100 Chinese yuan (3–13 US dollars) through online purchases or pharmaceutical stores. An understanding of migrant workers’ intention to get tested is necessary for targeted HIV control, prevention, and intervention promotion.

Across countries, studies have investigated HIV testing behaviors among external migrants who were not born in the study countries [29]. Results showed that 21–64% of these migrants had ever been tested for HIV [30,31,32,33,34], and 5.2–19.8% of them intended to do so in the next 12 months [35]. In China, there are few studies on HIV testing behaviors among internal migrants [36,37,38,39]. The proportion of those with a history of HIV testing ranged from 2.3% to 6.0% [36,37,38]. One study showed that 70.4% of Chinese migrant workers who were MSM had ever received HIV testing in their lifetime [39].

Previous studies revealed that certain sociodemographic variables were associated with the uptake of HIV testing. A higher likelihood of HIV testing was reported among young individuals [40], the unmarried [41], and those with lower incomes [42]. Studies have also shown ethnicity as a significant predictor of HIV testing, e.g., African Americans, when compared with white Americans, were more likely to report a previous HIV test [43]. In addition, education level has also been reported as a predictor, though with inconsistent findings [34,44]. With regard to drinking behavior, studies have found heavy drinkers to be more likely to engage in high-risk sexual behaviors [45,46] and less likely to be tested for HIV [45,47,48]. Therefore, these potentially vital determinants were also considered in this study.

The Health Belief Model (HBM) provided a theoretical framework to identify factors associated with the behavioral intention to take up HIV testing and HIVST in this study. It is one of the oldest yet most enduring models used in health promotion and disease prevention [49,50]. It has been widely used to explain and predict health-related behaviors, including HIV testing and HIVST [23,51,52,53,54,55]. The HBM has six constructs, namely, perceived susceptibility (the subjective belief that a person may acquire a disease or enter a harmful state), perceived severity (the belief in the extent of harm that can result from an acquired disease or harmful state as a result of a particular behavior), perceived benefits (beliefs in the efficacy of a target behavior in reducing the risk or seriousness of a health problem), perceived barriers (the belief concerning the costs of performing a suggested behavior), cues to action (precipitating force that makes a person feel the need to take action), and self-efficacy (the confidence in one’s ability to acquire a new behavior or change a behavior) [50]. In this study, these constructs were examined to see whether they were associated with the behavioral intention to take up HIV testing and HIVST.

It is essential to understand the various factors associated with behavioral intention to take up any type of HIV testing and HIVST in order to design effective health promotion measures. However, there is a lack of studies investigating factors related to behavioral intention toward the use of HIVST among migrant workers. To address the abovementioned knowledge gaps, by using secondary data analysis, this study investigated the prevalence of and behavioral intention to undergo HIV testing/HIVST in the next six months among male migrant workers with high-risk sexual behaviors in the past six months. The study used the HBM as a theoretical framework [49] to examine factors, both individual and interpersonal, associated with the behavioral intention to take up any form of testing.

## 2. Materials and Methods

### 2.1. Study Design

This study was a secondary data analysis of a cross-sectional study conducted in October and December 2019 among factory workers in Shenzhen, China [56]. As one of China’s major special economic zones, Shenzhen Municipality borders Hong Kong Special Administrative Region (SAR) to the south. The majority of the factories here are located in the Longhua district of Shenzhen, and there were over 1500 factories and more than one million factory workers in 2020 [57].

### 2.2. Participants and Data Collection

The participants in this study were full-time factory employees in Longhua district who were at least 18 years of age. A stratified multi-stage sampling method was used to recruit the participants [56]. There are 1805 factories listed in the most up-to-date registry kept by the Longhua CDC. The names of these factories were input into an Excel file. Using the function of “select random cells”, 16 factories were selected by the research team. These factories included four mechanical processing plants, three electronic device manufacturers, three printing and dyeing factories, two chemical raw material plants, one smelter, one garment factory, one food and beverage manufacturer, and one other factory. Within each participating factory, three to four workshops were then randomly selected. All full-time employees aged 18 years or above in the selected workshops were invited to participate in the study. A total of 2700 workers were approached, and 2023 completed the self-administered questionnaires [56]. Of 2023 completed records in the original dataset, we selected 363 subjects who were male internal migrants (i.e., without permanent residency of Shenzhen) having sexual intercourse with FSW and/or NRP in the past six months [4,5,10,11,12]. We defined a FSW as a woman who exchanged sex for money or gifts, and an NRP as a woman who was neither a FSW nor a participant’s stable girlfriend or wife. Ethics approval was obtained from the ethics committees of the School of Public Health, Sun Yat-sen University (2019/3).

### 2.3. Measurements

#### 2.3.1. Background Characteristics of the Participants

Information collected included social demographics, lifestyles, HIV/STI prevention service utilization, and sexual behaviors with FSW and NRP. Social demographics included age, ethnicity, relationship status, education level, whether living with a partner/spouse/children in Shenzhen, monthly personal income, and status as frontline workers or management staff. Ethnicity was classified into two categories, namely, Han majority and other ethnic minorities (there are 55 ethnic minority groups in China). Lifestyles included cigarette smoking in the past year and alcohol drinking status measured by the Alcohol Use Disorders Identification Test (AUDIT). HIV/STI prevention service utilization included participants’ use of any type of HIV testing and HIVST in their lifetimes and use of other HIV/STI prevention services (receiving free condoms and pamphlets and attending workshops/seminars) in the past six months. Sexual behaviors included whether they had sexual intercourse with RP, NRP, FSW, whether they had unprotected sex with RP, NRP, and FSW, and sexualized drug use (use of psychoactive substances before/during sexual intercourse).

#### 2.3.2. Uptake and Behavioral Intention to Take Up Any Type of HIV Testing and HIVST

Participants were asked to answer whether they had ever used any types of HIV testing and HIVST. Then they were asked whether they were willing to take up HIV testing and HIVST in the next 6 months (response categories: 1 = very unlikely, 2 = unlikely, 3 = neutral, 4 = likely, and 5 = very likely). Participants who selected “4” (likely) or “5” (very likely) were regarded as having a behavioral intention.

#### 2.3.3. Individual-Level Variables

Variables assessed at the individual level included perceptions related to HIV, HIV testing in general, and HIVST, based on the HBM. The Risk Perception Scale was developed by combining scores from two items measuring perceived risk of HIV and other STIs in the next six months (response categories: 1 = very low, 2 = low, 3 = neutral, 4 = high, and 5 = very high). The Cronbach’s alpha of the Risk Perception Scale was 0.90.

A 2-item Perceived Barrier Scale was developed by combining individual item scores of perceptions concerning HIV testing in general. The Cronbach’s alpha of the Perceived Barrier Scale was 0.64. In addition, three single items measured perceived benefit (i.e., taking up HIV testing could detect HIV infection earlier so as to have better treatment outcomes), perceived cue to action (i.e., people who are important to you suggest you take up HIV testing), and perceived self-efficacy of HIV testing in general (i.e., you are confident to take up HIV testing in the next six months). Responses for the abovementioned items were as follows: 1 = disagree, 2 = neutral, 3 = agree.

To assess perceptions specific to HIVST, validated scales among Chinese MSM were adapted in this study [53]. They included a 2-item Perceived Benefit Specific to HIVST Scale, a 5-item Perceived Barrier Specific to HIVST Scale, and 2 single items measuring perceived cue to action and self-efficacy specific to HIVST. The Cronbach’s alpha of these two scales was 0.63 and 0.76, respectively.

#### 2.3.4. Interpersonal-Level Variables

One item measured the frequency of exposure to health-related content on short video apps (e.g., TikTok, Kwai) and another item measured the frequency of exposure to content related to HIV and STI on short video apps (e.g., TikTok, Kwai). Response categories were as follows: 1 = never, 2 = seldom, 3 = sometimes, 4 = often.

### 2.4. Sample Size Planning

A total of 363 participants were included in this secondary analysis. The behavioral intention to take up any HIV testing/HIVST was 20–25%. Given a statistical power of 0.80 and alpha value of 0.05, and assuming the behavioral intention to take up any HIV testing/HIVST in the reference group (without a facilitating condition) to be 5–15%, this sample size could detect the smallest odds ratios of 2.09 between people with and without a facilitating condition (PASS 11.0, NCSS, LLC).

### 2.5. Statistical Analysis

Using logistic regression models, crude odds ratios (OR) were obtained considering behavioral intention to take up any type of HIV testing and HIVST in the next six months as dependent variables, and sociodemographic characteristics as independent variables. After adjusting for those sociodemographic characteristics with *p* < 0.05 in the univariate analysis, the associations between the dependent variables and independent variables of interest (e.g., perceptions related to HIV testing in general and/or perceptions specific to HIVST, and interpersonal variables) were then assessed and adjusted odds ratios (AOR) obtained. The 95% confidence intervals (CI) of the ORs and AORs were also obtained. In order to determine each AOR, a single logistic regression model was fitted, which included one of the independent variables and all the significant sociodemographic characteristics. SPSS 27 (IBM, Armonk, NY, USA) was used in data analysis, with *p*-values < 0.05 taken as indicating statistical significance.

## 3. Results

### 3.1. Background Characteristics of the Participants

Among 363 subjects, the majority were less than 40 years old (93.4%), of Han ethnicity (82.6%), and not registered permanent residents of Shenzhen (98.3%). About half did not receive senior high education (54.8%), and three-quarters were not living with a partner/spouse/children in Shenzhen (76.0%). In the past six months, 91.7% and 79.6% of the participants had sexual intercourse with NRP and FSW, respectively. The prevalence of condomless sex with NRP and FSW was 19.6% and 10.7%, respectively (Table 1).

### 3.2. History of HIV Testing/HIVST and Behavioral Intention to Take Up the Testing

Only 16.5% of the participants reported that they had used any type of HIV testing in their lifetimes, and 12.7% for HIVST (Table 1). Among the participants, 25.6% and 23.7% intended to take up any type of HIV testing and HIVST in the next six months, respectively (Table 2).

### 3.3. Associations between Background Characteristics and Behavioral Intention to Take Up Any Type of HIV Testing/HIVST in the Next Six Months

The associations between background characteristics and behavioral intention to take up any type of HIV testing were statistically non-significant. However, participants who had used other HIV/STI prevention services and scored 8–15 in the AUDIT (hazardous drinking) were associated with higher intentions to take up HIVST in the next six months (Table 3).

### 3.4. Factors Associated with Behavioral Intention to Take Up Any Type of HIV Testing and HIVST in the Next Six Months

For behavioral intention to take up any type of HIV testing, as there were no statistically significant background characteristics; only a univariate analysis was conducted. The results showed that perceived benefits (OR: 2.29, 95% CI: 1.53, 3.41), perceived cue to action (OR: 2.34, 95% CI: 1.61, 3.41), perceived self-efficacy (OR: 2.99, 95% CI: 2.03, 4.39) related to HIV testing in general, and frequency of exposure to health-related content (OR: 1.57, 95% CI: 1.19, 2.05) and HIV or STI-related content (OR: 1.41, 95% CI: 1.06, 1.87) on short video apps were associated with higher behavioral intentions to take up any type of HIV testing in the next six months. Perceived barrier to receive HIV testing in general was, however, negatively associated with the dependent variable (OR: 0.78, 95% CI: 0.64, 0.95) (Table 4).

**Figure 1:**
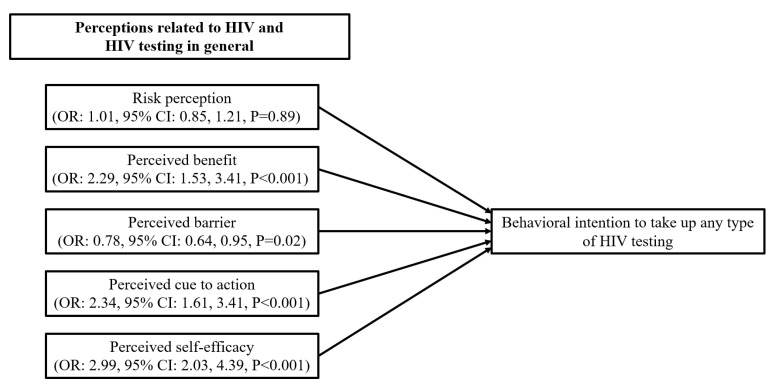
Factors associated with behavioral intention to take up any type of HIV testing.

**Figure 2:**
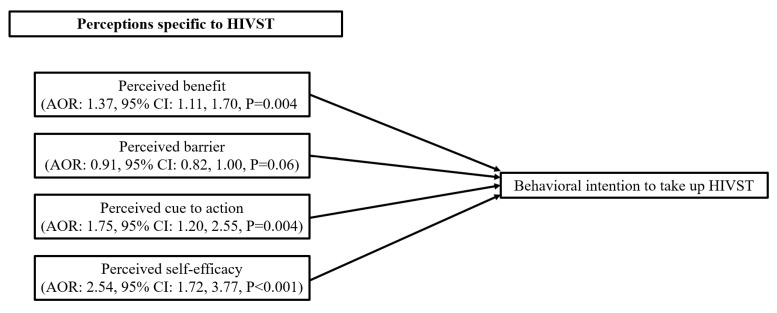
Factors associated with behavioral intention to take up HIVST.

For behavioral intention to take up HIVST, after adjusting for significant background characteristics, perceived benefit (AOR: 2.00, 95% CI: 1.33, 3.00), perceived cue to action (AOR: 2.13, 95% CI: 1.44, 3.14) and perceived self-efficacy (AOR: 2.94, 95% CI: 1.96, 4.42) related to HIV testing in general, and perceived benefit (AOR: 1.37, 95% CI: 1.11, 1.70), perceived cue to action (AOR: 1.75, 95% CI: 1.20, 2.55), and perceived self-efficacy specific to HIVST (AOR: 2.54, 95% CI: 1.72, 3.77), as well as frequency of exposure to content related to health (AOR: 1.77, 95% CI: 1.32, 2.36) and HIV/STI (AOR: 1.42, 95% CI: 1.04, 1.93) on short video apps were significantly associated with behavioral intentions to take up HIVST in the next six months (Table 4). The results are also shown in Figure 1 and Figure 2.

## 4. Discussion

This is one of the first studies examining behavioral intention to take up any type of HIV testing and HIVST and the associated factors among migrant male factory workers in China. Factors at the individual and interpersonal levels as determinants of HIV testing were considered. The findings provided a knowledge basis to develop tailored behavioral interventions to increase the uptake of HIV testing among migrant male factory workers.

The lifetime uptake of any type of HIV testing (16.5% vs. 60.5%) and HIVST (12.7% vs. 26.2%) among high-risk migrant workers was much lower than that of MSM in China [58,59]. Moreover, less than one quarter of the study sample intended to take up any type of HIV testing or HIVST in future. There is hence much room for improvement. Some factors may contribute to the discrepancy in lifetime uptake and low behavioral intentions between male migrant workers and MSM. First, according to previous research, many migrant workers lacked HIV/STI-related knowledge., and about 40% did not know that using condoms would decrease the risk of HIV infection, indicating insufficient awareness of self-protection against HIV infection [60]. In contrast, MSM in China are knowledgeable about HIV and STIs [61,62]. Second, although HIV-related programs in key populations are large-scale with strong governmental support in China [63], migrant workers are not one of the target populations. These programs focus on MSM and FSW [2]. Without actively promoting HIV testing and HIVST among such underlooked high-risk populations and providing comprehensive support services, migrant workers may not have the intention to do so.

Since barriers and facilitators to receive any type of HIV testing and HIVST were found, the findings provided some empirical insights for developing interventions promoting HIV testing. The HBM is a potentially useful framework to guide the development of future programs promoting HIV testing. Perceived benefit, perceived cue to action, and perceived self-efficacy were associated with higher behavioral intention to take up any type of HIV testing, while perceived barriers were negatively associated with the dependent variable. Over half of participants (60.9%) perceived some benefits of HIV testing in general, and many believed it could detect HIV infection earlier so as to enable better treatment outcomes. Those who scored higher in perceived benefit of HIV testing were more likely to have higher behavioral intentions to take up any types of HIV testing in the next six months. Future health promotion campaigns should strengthen such beliefs among migrant factory workers. In the health promotion campaigns, some information on the importance of early HIV testing/detection to treatment outcomes should be provided. About 21.2% of participants had concerns that others would think they had high-risk sexual behaviors if they took up HIV testing and the location/working hours of the HIV testing service providers was inconvenient for them. It is important to address such concerns among migrant factory workers, as they were significantly associated with lower intentions to take up HIV testing. Service providers should make migrant factory workers feel that their privacy is well protected when taking up HIV testing. Additionally, service providers could include HIV testing as one of the components in a general body check scheme to avoid stigmatization, unlike taking HIV testing only. For locations and service hours, providing HIV testing services near the factories and extending service hours until late at night, as most migrant factory workers work during daytime hours, could be considered. Perceived cue to action and self-efficacy were both facilitators. Future programs should consider and use significant others who are also migrant factory workers (e.g., peers, relatives) to give reminders to others take up HIV testing as a strong cue to action. For instance, promotional videos should include these significant others who have already taken up an HIV test talking about the importance and benefits of early detection. To increase self-efficacy, creating promotional videos with a role model (i.e., migrant workers) demonstrating the specific procedures to take up HIV testing would be beneficial. The specific procedures should include where to take an HIV test, how to make an appointment, how long it will take to have the test results and so on. Furthermore, a role model can demonstrate how to form an action plan to take up HIV testing, which is also a potentially useful strategy.

For constructs specific to HIVST, over a quarter of participants (27.8%) perceived the benefits of using HIVST as it could avoid being stigmatized by HIV testing service providers and about one fifth (20.9%) thought that using HIVST was convenient to them. Future health promotion programs should enhance such beliefs among migrant factory workers. For instance, promotional videos should demonstrate how to order an HIVST kit, how to use it, how to read the test results, and how to seek help if tested positive. Providing a strong cue to action to take up HIVST and improving self-efficacy in taking up HIVST are also useful strategies to promote HIVST among migrant factory workers. Similar strategies suggested for HIV testing should also be useful for HIVST among migrant factory workers.

Furthermore, our findings revealed that people with a high frequency of exposure to health-related content on short video apps (e.g., TikTok, Kwai) and content related to HIV and STI on those apps had a higher behavioral intention to take up HIV testing or HIVST. Around 40 million people use TikTok every day [64]. Service providers may consider promoting HIV testing or HIVST targeting migrant workers on these short video apps, such as through key leaders with whom the migrant factory workers are familiar with.

This study had several limitations. First, causal relationships could not be established, as our study design was cross-sectional and based on secondary data analysis. Second, the data were self-reported, and social desirability bias might exist. The prevalence of sexual risk behaviors might be under-reported, whereas behavioral intention to take up any type of HIV testing or HIVST might be over-reported, although anonymity should have reduced such biases. Finally, this study was conducted in one Chinese city and the participants could not represent all migrant workers in China. Therefore, the generalization of the results to other parts of China should be made with caution. Despite the limitations, the study provided useful insight on HIV testing behavior among migrant factory workers in China, and can be used as a basis for further studies on the same topic.

## 5. Conclusions

In conclusion, the uptake and behavioral intention to take up any type of HIV testing and HIVST was low among high-risk male migrant workers in China. Perceptions related to HIV and HIV testing in general based on the HBM and frequency of exposure to content related to health and/or HIV/STI on short videos apps were significant determinants of behavioral intention to take up any type of HIV testing. In addition, perceptions specific to HIVST were associated with behavioral intention to use HIVST. This study provided practical implications for designing interventions to increase HIV testing and HIVST among migrant workers.

## Figures and Tables

**Table 1:** Background characteristics of the participants (n = 363).

	N	%
**Sociodemographic Characteristics**		
Age group (years)		
18–30	189	52.1
31–40	150	41.3
41–50	19	5.2
>50	5	1.4
Ethnicity		
Han majority	300	82.6
Other ethnic minorities	63	17.4
Relationship status		
Currently single	177	48.8
Having a regular partner/stable girlfriend	42	11.6
Married or cohabited with a woman	111	30.6
Divorced, separated, or widowed	16	4.4
Education level		
Junior high or below	199	54.8
Senior high or equivalent	133	36.6
College/university and above	23	6.3
Living with a partner/spouse/children in Shenzhen		
No	276	76.0
Yes	87	24.0
Monthly personal income, China Yuan (USD)		
<3000 (469.8)	32	8.8
3000–4999 (469.8)	219	60.3
5000–9999 (782.9–1565.7)	97	26.7
≥10,000	4	1.1
Status as frontline workers or management staff		
Frontline workers	291	80.2
Management staff	72	19.8
**Lifestyles**		
Alcohol drinking status (score of Alcohol Use Disorders Identification Test, AUDIT)		
Low-risk consumption (<8)	243	66.9
Hazardous drinking (8–15)	99	27.3
Harmful drinking (16–19)	13	3.6
Probable alcohol dependence (20–40)	8	2.2
Cigarette smoking in the past year		
No	165	45.5
Yes	198	54.5
**HIV/STI prevention service utilization**		
Use of any type of HIV testing in lifetime		
No	303	83.5
Yes	60	16.5
Use of HIVST in lifetime		
No	317	67.3
Yes	46	12.7
Use of other HIV/STI prevention services (receiving free condoms and pamphlets, and attending workshops/seminars) in the past six months		
No/uncertain	275	75.8
Yes	88	24.2
Sexual behaviours in the past six months		
Sexual intercourse with regular female sex partners (RP)		
No	33	9.1
Yes	330	90.9
Sexual intercourse with non-regular female sex partners (NRP)		
No	30	8.3
Yes	333	91.7
Sexual intercourse with female sex workers (FSW)		
No	74	20.4
Yes	289	79.6
Condomless sex with RP		
No	290	79.9
Yes	73	22.1
Condomless sex with NRP		
No	292	80.4
Yes	71	19.6
Condomless sex with FSW		
No	324	89.3
Yes	39	10.7
Sexualized drug use (use of psychoactive substance before/during sexual intercourse)		
No	360	99.2
Yes	3	0.8

**Table 2:** Behavioral intention to use any type of HIV testing and HIVST, and descriptive data of variables at the individual and interpersonal levels (n = 363).

	N	%
**Behavioral intention to take up HIV testing in the next six months**		
Likelihood of receiving any type of HIV testing		
Very unlikely	110	30.3
Unlikely	58	16.0
Neutral	102	28.1
Likely	74	20.4
Very likely	19	5.2
Likelihood of receiving HIVST		
Very unlikely	106	29.2
Unlikely	75	20.7
Neutral	96	26.4
Likely	76	20.9
Very likely	10	2.8
Individual-level factors		
**Perceptions related to HIV and HIV testing in general**		
Perceived risk of contracting HIV/STI, n (%) high/very high		
Perceived chance of contracting HIV in the next six months	4	1.2
Perceived chance of contracting STI in the next six months	3	0.9
Risk Perception Scale ^1^		
Scale score, mean (SD)	2.6	(1.3)
Perceived benefit of HIV testing in general, n (%) agree		
Taking up HIV testing could detect HIV infection earlier so as to enable better treatment outcomes	221	60.9
Item score, mean (SD)	2.4	(0.8)
Perceived barrier to receive HIV testing in general, n (%) agree		
Others would think you have high-risk sexual behaviors if you take up HIV testing	77	21.2
Location and/or working hours of organizations providing HIV testing is inconvenient for you	83	22.9
Perceived Barrier Scale ^2^		
Scale score, mean (SD)	3.9	(1.2)
Perceived cue to action, n (%) agree		
People who are important to you suggest that you take up HIV testing	116	32.0
Item score, mean (SD)	2.1	(0.7)
Perceived self-efficacy, n (%) agree		
You are confident to take up HIV testing in the next six months	108	29.8
Item score, mean (SD)	2.1	(0.7)
**Perceptions specific to HIVST**		
Perceived benefit of HIVST, n (%) agree		
HIVST is very convenient for you	76	20.9
Using HIVST can prevent being stigmatized by HIV testing service providers	101	27.8
Perceived Benefit Specific to HIVST Scale ^3^		
Scale score, mean (SD)	4.0	(1.2)
Perceived barrier specific to HIVST, n (%) agree		
You are concerned about the accuracy of HIVST	71	19.6
You find it hard to interpret the results of HIVST	95	26.2
You are concerned that others would find out you are using HIVST	87	24.0
If tested to be positive by HIVST, you would not know what to do	76	20.9
The cost of an HIVST kit (20–100 China Yuan or 3–13 USD) is too expensive for you	75	20.7
Perceived Barrier Specific to HIVST Scale ^4^		
Scale score, mean (SD)	9.72	2.52
Perceived cue to action specific to HIVST, n (%) agree		
People who are important to you suggest that you take up HIVST	124	34.2
Item score, mean (SD)	2.2	(0.7)
Perceived self-efficacy specific to HIVST, n (%) agree		
You are confident to take up HIVST in the next six months	116	32.0
Item score, mean (SD)	2.1	(0.7)
**Interpersonal-level factors**		
Frequency of exposure to health-related content on short video apps (e.g., TikTok, Kwai)		
Never	67	18.5
Seldom	156	43.0
Sometimes	104	28.7
Often	36	9.9
Frequency of exposure to content related to HIV and STI on short video apps (e.g., TikTok, Kwai)		
Never	135	37.2
Seldom	149	41.0
Sometimes	69	19.0
Often	10	2.8

^1^ Risk Perception Scale, two items, Cronbach’s alpha: 0.90; ^2^ Perceived Barrier Scale, two items, Cronbach’s alpha: 0.64; ^3^ Perceived Benefit Specific to HIVST Scale, two items, Cronbach’s alpha: 0.63; ^4^ Perceived Barrier Specific to HIVST Scale, five items, Cronbach’s alpha: 0.76.

**Table 3:** Associations between background characteristics and behavioral intention to take up any types of HIV testing/HIVST in the next six months (n = 363).

	Behavioral Intention to Take Up Any Type of HIV Testing in the Next Six Months		Behavioral Intention to Take Up HIVST in the Next Six Months	
	OR (95% CI)	*p* Value	OR (95% CI)	*p* Value
**Sociodemographic Characteristics**				
Age group (years)				
18–30	1.0		1.0	
31–40	0.97 (0.59, 1.58)	0.90	0.98 (0.60, 1.62)	0.94
41–50	1.32 (0.48, 3.66)	0.60	1.83 (0.26, 2.62)	0.75
>50	N.A.	N.A.	N.A.	N.A.
Ethnicity				
Han majority	1.0		1.0	
Other ethnic minorities	0.80 (0.42, 1.53)	0.50	1.36 (0.74, 2.51)	0.32
Relationship status				
Currently single	1.0		1.0	
Have a regular partner/stable girlfriend	1.36 (0.65, 2.83)	0.42	1.51 (0.71, 3.24)	0.29
Married or cohabited with a woman	0.97 (0.56, 1.69)	0.92	1.40 (0.80, 2.44)	0.23
Divorced, separated, or widowed	1.81 (0.62, 5.68)	0.28	2.27 (0.78, 6.65)	0.14
Education level				
Junior high or below	1.0		1.0	
Senior high or equivalent	0.93 (0.56, 1.54)	0.79	1.21 (0.72, 2.02)	0.47
College/university and above	0.78 (0.28, 2.22)	0.65	1.24 (0.24, 3.34)	0.67
Living with a partner/spouse/children in Shenzhen				
No	1.0			
Yes	1.14 (0.66, 1.97)	0.63	1.22 (0.70, 2.11)	0.49
Monthly personal income, China Yuan (US$)				
<3000 (469.8)	1.0		1.0	
3000–4999 (469.8)	1.06 (0.43, 2.59)	0.90	1.28 (0.50, 3.29)	0.60
5000–9999 (782.9–1565.7)	1.68 (0.66, 4.30)	0.28	1.59 (0.59, 4.29)	0.36
≥10,000 (1566)	3.57 (0.42, 30.10)	0.24	1.44 (0.13, 16.42)	0.77
Status as frontline workers or management staff				
Frontline workers	1.0		1.0	
Management staff	1.25 (0.71, 2.22)	0.44	0.90 (0.49, 1.67)	0.74
**Lifestyles**				
Alcohol drinking status (score of Alcohol Use Disorders Identification Test, AUDIT)				
Low-risk consumption (<8)	1.0		1.0	
Hazardous drinking (8–15)	1.13 (0.67, 1.90)	0.66	1.84 (1.09, 3.11)	0.02
Harmful drinking (16–19)	N.A.	N.A.	0.32 (0.04, 2.53)	0.28
Probable alcohol dependence (20–40)	0.95 (0.19, 4.84)	0.95	2.32 (0.54, 10.02)	0.26
Cigarette smoking in the past year				
No	1.0		1.0	
Yes	1.02 (0.63, 1.63)	0.95	1.37 (0.84, 2.24)	0.21
**HIV/STI prevention service utilization**				
Use of any type of HIV testing in lifetime				
No	1.0		1.0	
Yes	0.86 (0.35, 1.36)	0.28	1.21 (0.64, 2.28)	0.55
Use of HIVST in lifetime				
No	1.0			
Yes	0.68 (0.31, 1.46)	0.32	1.32 (0.66, 2.63)	0.44
Use of other HIV/STI prevention services (receiving free condoms and pamphlets, and attending workshops/seminars) in the past six months				
No/uncertain	1.0		1.0	
Yes	1.40 (0.82, 2.38)	0.21	1.75 (1.02, 2.98)	0.04
**Sexual behaviors in the past six months**				
Sexual intercourse with regular female sex partners (RP)				
No	1.0		1.0	
Yes	1.08 (0.47, 2.50)	0.85	1.17 (0.49, 2.80)	0.73
Sexual intercourse with non-regular female sex partners (NRP)				
No	1.0		1.0	
Yes	0.66 (0.30, 1.48)	0.32	1.02 (0.42, 2.47)	0.96
Sexual intercourse with female sex workers (FSW)				
No	1.0		1.0	
Yes	1.09 (0.60, 1.97)	0.78	0.87 (0.48, 1.57)	0.65
Condomless sex with RP				
No	1.0		1.0	
Yes	1.33 (0.76, 2.35)	0.32	1.28 (0.72, 2.29)	0.41
Condomless sex with NRP				
No	1.0			
Yes	0.81 (0.44, 1.50)	0.51	0.92 (0.50, 1.71)	0.80
Condomless sex with FSW				
No	1.0			
Yes	0.86 (0.39, 1.88)	0.70	0.96 (0.44, 2.12)	0.92
Sexualized drug use (use of psychoactive substances before/during sexual intercourse)				
No	1.0		1.0	
Yes	N.A.	N.A.	N.A.	N.A.

OR: crude odds ratios, CI: confidence interval, N.A.: not applicable.

**Table 4:** Factors associated with behavioral intentions to take up any type of HIV testing and HIVST.

	Behavioral Intention to Take Up Any Type of HIV Testing		Behavioral Intention to Take Up HIVST	
	OR (95% CI)	*p* Value	AOR (95% CI)	*p* Value
**Perceptions related to HIV and HIV testing in general**				
Risk Perception Scale	1.01 (0.85, 1.21)	0.89	0.99 (0.82, 1.19)	0.89
Perceived benefit of HIV testing in general	2.29 (1.53, 3.41)	<0.001	2.00 (1.33, 3.00)	0.001
Perceived Barrier Scale	0.78 (0.64, 0.95)	0.02	1.17 (0.96, 1.44)	0.13
People who are important to you suggest that you take up HIV testing (perceived cue to action)	2.34 (1.61, 3.41)	<0.001	2.13 (1.44, 3.14)	<.001
You are confident to take up HIV testing in the next six months (perceived self-efficacy)	2.99 (2.03, 4.39)	<0.001	2.94 (1.96, 4.42)	<0.001
**Perceptions specific to HIVST**				
Perceived Benefit Specific to HIVST Scale	N.A.	N.A.	1.37 (1.11, 1.70)	0.004
Perceived Barrier Specific to HIVST Scale	N.A.	N.A.	0.91 (0.82, 1.00)	0.06
People who are important to you suggest that you take up HIVST (perceived cue to action)	N.A.	N.A.	1.75 (1.20, 2.55)	0.004
You are confident to take up HIVST in the next six months (perceived self-efficacy)	N.A.	N.A.	2.54 (1.72, 3.77)	<0.001
**Interpersonal-level variables**				
Frequency of exposure to health-related content on short video apps (e.g., TikTok, Kwai)	1.57 (1.19, 2.05)	0.001	1.77 (1.32, 2.36)	<0.001
Frequency of exposure to content related to HIV and STI on short video apps (e.g., TikTok, Kwai)	1.41 (1.06, 1.87)	0.02	1.42 (1.04, 1.93)	0.02

OR: crude odds ratios; AOR: adjusted odds ratios, as adjusted for significant background characteristics listed in Table 3; N.A.: not applicable.

## Data Availability

The data presented in this study are available from the corresponding author upon request. The data are not publicly available as they contain personal behaviors.
